# Genetic diversity and recombination of enterovirus G strains in Japanese pigs: High prevalence of strains carrying a papain-like cysteine protease sequence in the enterovirus G population

**DOI:** 10.1371/journal.pone.0190819

**Published:** 2018-01-11

**Authors:** Shinobu Tsuchiaka, Yuki Naoi, Ryo Imai, Tsuneyuki Masuda, Mika Ito, Masataka Akagami, Yoshinao Ouchi, Kazuo Ishii, Shoichi Sakaguchi, Tsutomu Omatsu, Yukie Katayama, Mami Oba, Junsuke Shirai, Yuki Satani, Yasuhiro Takashima, Yuji Taniguchi, Masaki Takasu, Hiroo Madarame, Fujiko Sunaga, Hiroshi Aoki, Shinji Makino, Tetsuya Mizutani, Makoto Nagai

**Affiliations:** 1 Research and Education Center for Prevention of Global Infectious Disease of Animal, Tokyo University of Agriculture and Technology, Fuchu, Tokyo, Japan; 2 Kurayoshi Livestock Hygiene Service Center, Kurayoshi, Tottori, Japan; 3 Ishikawa Nanbu Livestock Hygiene Service Center, Kanazawa, Ishikawa, Japan; 4 Kenpoku Livestock Hygiene Service Center, Mito, Ibaraki, Japan; 5 Department of Veterinary Medicine, Faculty of Applied Biological Sciences, Gifu University, Yanagido, Gifu, Japan; 6 Education and Research Center for Food Animal Health, Gifu University (GeFAH), Gifu, Japan; 7 Center for Highly Advanced Integration of Nano and Life Sciences, Gifu University (G-CHAIN), Gifu, Japan; 8 Laboratory of Small Animal Clinics, School of Veterinary Medicine, Azabu University, Sagamihara, Kanagawa, Japan; 9 Department of Veterinary Medicine, School of Veterinary Medicine, Azabu University, Sagamihara, Kanagawa, Japan; 10 Faculty of Veterinary Science, Nippon Veterinary and Life Science University, Musashino, Tokyo, Japan; 11 Department of Microbiology and Immunology, The University of Texas Medical Branch at Galveston, Galveston, Texas, United States of America; 12 Department of Bioproduction Science, Ishikawa Prefectural University, Nonoichi, Ishikawa, Japan; University of Hong Kong, HONG KONG

## Abstract

To study the genetic diversity of enterovirus G (EV-G) among Japanese pigs, metagenomics sequencing was performed on fecal samples from pigs with or without diarrhea, collected between 2014 and 2016. Fifty-nine EV-G sequences, which were >5,000 nucleotides long, were obtained. By complete VP1 sequence analysis, Japanese EV-G isolates were classified into G1 (17 strains), G2 (four strains), G3 (22 strains), G4 (two strains), G6 (two strains), G9 (six strains), G10 (five strains), and a new genotype (one strain). Remarkably, 16 G1 and one G2 strain identified in diarrheic (23.5%; four strains) or normal (76.5%; 13 strains) fecal samples possessed a papain-like cysteine protease (PL-CP) sequence, which was recently found in the USA and Belgium in the EV-G genome, at the 2C–3A junction site. This paper presents the first report of the high prevalence of viruses carrying PL-CP in the EV-G population. Furthermore, possible inter- and intragenotype recombination events were found among EV-G strains, including G1-PL-CP strains. Our findings may advance the understanding of the molecular epidemiology and genetic evolution of EV-Gs.

## Introduction

Porcine enteroviruses (PEVs), members of the family *Picornaviridae*, are positive-sense, single-stranded nonenveloped RNA viruses, whose genomes consist of a single, large open reading frame encoding a single polyprotein flanked by 5′ and 3′ untranslated regions (5′UTR and 3′UTR) and a poly(A) tail at its 3′ end [1]. PEVs were originally classified into 13 types (PEV-1 to PEV-13) on the basis of virus neutralization assay results [2–3]. After further genomic studies, PEV-1 to -7 and PEV-11 to -13 have been reclassified and assigned to the genus *Teschovirus* and PEV-8, formally belonging to PEV-A, has been renamed porcine sapelovirus 1 and reclassified into the genus *Sapelovirus* [1, 4–6]. PEV-B, consisting of PEV-9 and -10, was reclassified as enterovirus G (EV-G). PEV-9 and -10, the prototypical EV-Gs isolated in 1973 and 1975 in UK, were renamed as EV-G1 and EV-G2, respectively [1, 7]. Currently, 16 EV-G genotypes are known to exist in Hungary, South Korea, the USA, China, Vietnam, and Belgium [8–15].

Although porcine teschoviruses and sapeloviruses have been identified as the cause of occasional diverse disorders, including gastrointestinal diseases, polioencephalomyelitis, and respiratory diseases [16–22], clinical relevance of EV-Gs to enteric or other disorders—apart from cases of skin lesion, pyrexia, and flaccid paralysis—has not been elucidated [7, 23]. Very recently, the unique EV-Gs that have a papain-like cysteine protease sequence (PL-CP) in the 2C–3A junction region of their genomes were identified in fecal samples from three independent cases of porcine diarrhea in the USA and Belgium [24–26]. The PL-CP has sequence identity to that of toroviruses (which are members of the order *Nidovirales*), in the ORF1a region. The PL-CP of nidoviruses acts as a protease to cleave peptide bonds and as a deubiquitinase to cleave the isopeptide bonds in polyubiquitin chains [27–29]. Viral deubiquitinases can remove the protective effect of attached ubiquitin-like molecules such as the protein encoded by interferon stimulated gene 15. These viral protease and deubiquitinase activities can modulate or block activation of the innate immune response [29–31]. EV-G PL-CP also shows strong deubiquitination and deISGylation activities and is thought to influence enteroviral genome plasticity and viral pathogenesis by acting as an innate-immunity antagonist [25].

In the present study, we investigated the genetic diversity of EV-G isolates from fecal samples from pigs in Japan via the metagenomics approach. We detected high genetic diversity of Japanese EV-Gs and high prevalence of viruses carrying PL-CP in the EV-G population for the first time. Furthermore, possible inter- and intragenotype recombination events were found in the EV-G strains, including G1-PL-CP strains.

## Materials and methods

### Samples, cDNA construction, and next-generation sequencing

A total of 222 fecal samples from 6- to 100-day-old pigs from 77 pig farms, including 126 normal feces, 21 feces with mild diarrhea, and 75 diarrheic feces, were subjected to metagenomics analysis. cDNA libraries were constructed as previously described [32]. Briefly, total RNA was extracted directly from the supernatant of a 10% fecal suspension by means of the TRIzol LS Reagent (Life Technologies, Carlsbad, CA, USA) and treated with DNase I (Takara Bio, Shiga, Japan). cDNA libraries for next-generation sequencing were built using the NEBNext Ultra RNA Library Prep Kit for Illumina (New England Biolabs, Ipswich, MA, USA), according to the manufacturer’s instruction. Sequencing was carried out on a MiSeq bench-top sequencer (Illumina, San Diego, CA, USA), using 151 pair-end reads. Contigs were generated from trimmed sequence reads by *de novo* assembly, and the generated contigs were evaluated by means of mapping reads to a reference command in CLC Genomics Workbench with strictest parameter settings (mismatch cost, 2; insertion cost, 3; deletion cost, 3; length function, 0.9; and similarity function, 0.9), and 5′ and 3′ sequences with insufficient read depth (<3) were discarded.

### Genome analysis

The nucleotide (nt) and amino acid (aa) sequences were aligned in the ClustalW software [33] followed by phylogenetic analysis via the maximum-likelihood method in the MEGA 5.22 software [34]. The best-fit models in MEGA 5.22 were the GTR+G+I model for VP1, VP4-VP3, P2, and P3 phylogenetic trees and the WAG+G model for the PL-CP phylogenetic tree. Reliability of the phylogenetic trees was evaluated by bootstrap analysis with 1000 replicates [35]. Pairwise sequence identities were calculated using CLC Genomics Workbench 7.5.5 (CLC bio). Recombination analysis was conducted using the SimPlot software v.3.5.1 [36] and Recombination Detection Program 4 (RDP) [37].

### Ethics statement

Because the fecal samples were collected from naturally infected animals in the field, no specific approval was needed. Before starting work on this study, we contacted the farm owners and obtained their permission.

## Results

### Metagenomics analysis and EV-G detection

Next-generation sequencing was conducted on cDNA libraries constructed from total RNA of 222 fecal samples. Via a BLAST search, 59 EV-G-like contigs that were longer than 5,000 nt, including the entire VP1-coding sequence with more than threefold coverage of sequence reads, were identified in 35 (15.8%) normal fecal samples, five (2.3%) fecal samples for mild diarrhea, and 10 (4.5%) diarrheic fecal samples (Tables 1 and 2). Eight of 50 samples revealed more than two EV-G-like contigs (Table 1). Apart from three samples (Ishi-Ya3, Ishi-Ka3, and Ishi-Ka7), 47 samples were found to contain other viruses: Rotaviruses A, C, or H; orthoreovirus; kobuvirus; picobirnavirus; astrovirus; porcine epidemic diarrhea virus (PEDV); posavirus; sapelovirus; St-Valerien virus; sapovirus; or teschovirus (Table 1 and S3 Table).

**Table 1:** Information on EV-G-positive fecal samples from pigs in Japan.

Sample name	Collection year	Age of pigs (days)	Health status	Region (geographic coordinates)	Sample status	Number of EV-G contigs (Enterovirus G genotype)	Co-infection with other viruses
**Bu3-4**	2014	12	Without diarrhea	Niigata (37.902458,139.023407)	Single	1 (G3)	Rotavirus A (G4P[19]), Orthoreovirus
**Bu3-5**	2014	10	Without diarrhea	Niigata (37.902458,139.023407)	Single	1 (G3)	Rotavirus A (G4P[19]), Kobuvirus
**Bu3-6**	2014	6	Without diarrhea	Niigata (37.902458,139.023407)	Single	1 (G3)	Rotavirus A (G4P[19])
**Bu3-7**	2014	6	Without diarrhea	Niigata (37.902458,139.023407)	Single	1 (G3)	Rotavirus A (G4P[19])
**Bu4-1**	2014	21	Without diarrhea	Niigata (37.902458,139.023407)	Single	1 (G3)	Picobirnavirus
**Bu4-2**	2014	14	Without diarrhea	Niigata (37.902458,139.023407)	Single	1 (G3)	Astrovirus, Kobuvirus
**Bu4-4**	2014	16	Without diarrhea	Niigata (37.902458,139.023407)	Single	1 (G3)	Rotavirus A (G9P[13]), Astrovirus, Kobuvirus
**Bu4-6**	2014	20	Without diarrhea	Niigata (37.902458,139.023407)	Single	1 (G3)	Rotavirus A (G9P[13])
**Bu5-1**	2014	9	Without diarrhea	Tochigi (36.564579,139.883392)	Single	1 (G3)	Rotavirus A (G5P[23])
**Bu5-6**	2014	22	Mild diarrhea	Tochigi (36.564579,139.883392)	Single	1 (G3)	Kobuvirus
**Bu6-5**	2014	8	Diarrhea	Fukushima (37.750918,140.467823)	Single	1 (G3)	Rotavirus A (G9P[23])
**Bu8-2**	2014	26	Without diarrhea	Chiba (35.604561,140.123108)	Single	1 (G3)	Rotavirus A (G4P[6])
**Bu8-4**	2014	25	Diarrhea	Chiba (35.604561,140.123108)	Single	1 (G3)	Rotavirus A (G4P[6]), Picobirnavirus
**Iba26-489**	2014	<100	Diarrhea	Ibaraki (36.344040,140.445465)	Single	1 (G9)	Porcine epidemic diarrhea virus, Sapelovirus, Posavirus, Picobirnavirus
**Iba26-506**	2014	<100	Diarrhea	Ibaraki (36.344040,140.445465)	Single	1 (G2)	Porcine epidemic diarrhea virus, Posavirus,
**HgOg2-2**	2015	60	Without diarrhea	Tottori (35.503479,134.238266)	Single	1 (G1-PL-CP)	Astrovirus, Sapelovirus
**HgOg2-3**	2015	60	Without diarrhea	Tottori (35.503479,134.238266)	Single	1 (G1-PL-CP)	Astrovirus, Posavirus
**HgOg2-4**	2015	60	Without diarrhea	Tottori (35.503479,134.238266)	Single	2 (G1-PL-CP, G2)	Astrovirus
**HgOg2-5**	2015	60	Without diarrhea	Tottori (35.503479,134.238266)	Single	1 (G1-PL-CP)	Sapelovirus
**HgTa2-1**	2015	60	Without diarrhea	Tottori (35.503479,134.238266)	Single	2 (G1-PL-CP, G6)	Astrovirus, Rotavirus C, Sapelovirus
**HgTa2-2**	2015	60	Without diarrhea	Tottori (35.503479,134.238266)	Single	2 (G1-PL-CP, G9)	Astrovirus, Picobirnavirus, Sapovirus
**HgTa2-5**	2015	60	Without diarrhea	Tottori (35.503479,134.238266)	Single	1 (G1-PL-CP)	Astrovirus
**MoI2-1**	2015	60	Without diarrhea	Tottori (35.503479,134.238266)	Single	1 (G1-PL-CP)	Astrovirus, Sapovirus, Rotavirus A (G9P[13]), Porcine picornavirus Japan
**MoI2-2**	2015	60	Without diarrhea	Tottori (35.503479,134.238266)	Single	1 (G1-PL-CP)	Astrovirus, Sapelovirus, Teschovirus, Rotavirus C
**MoI2-3**	2015	60	Without diarrhea	Tottori (35.503479,134.238266)	Single	1 (G1-PL-CP)	Astrovirus, Rotavirus C
**HgYa2-1**	2015	60	Without diarrhea	Tottori (35.503479,134.238266)	Single	1 (G2-PL-CP)	Astrovirus, Sapelovirus, Torovirus, Rotavirus C
**HgYa2-3**	2015	60	Without diarrhea	Tottori (35.503479,134.238266)	Single	1 (G10)	Astrovirus, Sapelovirus, Picobirnavirus
**HgYa2-4**	2015	60	Without diarrhea	Tottori (35.503479,134.238266)	Single	1 (G10)	Astrovirus, Sapelovirus, Picobirnavirus
**Iba27-20**	2015	<100	Diarrhea	Ibaraki (36.344040,140.445465)	Single	1 (G9)	Porcine epidemic diarrhea virus, Picobirnavirus
**Iba27-21**	2015	<100	Diarrhea	Ibaraki (36.344040,140.445465)	Single	1 (G9)	Porcine epidemic diarrhea virus
**Iba27-107**	2015	<100	Diarrhea	Ibaraki (36.344040,140.445465)	Single	1 (G1-PL-CP)	Porcine epidemic diarrhea virus, Rotavirus A (G5P[13])
**Iba464-3**	2015	30	Diarrhea	Ibaraki (36.344040,140.445465)	Single	2 (G1, G4)	Astrovirus
**Iba464-4**	2015	30	Diarrhea	Ibaraki (36.344040,140.445465)	Single	2 (G1-PL-CP, G6)	Astrovirus, Rotavirus H
**Ishi-Sa4**	2015	20	Mild diarrhea	Ishikawa (36.595242,136.625671)	Pooled	1 (G3)	Rotavirus A (G9P[23]), Kobuvirus, Picobirnavirus
**Ishi-Sa5**	2015	20	Without diarrhea	Ishikawa (36.595242,136.625671)	Pooled	1 (G3)	Rotavirus C, Kobuvirus
**Ishi-Ya5**	2015	100	Diarrhea	Ishikawa (36.595242,136.625671)	Single	1 (G1-PL-CP)	St-Valerien swine virus, Sapelovirus
**Ishi-Ka3**	2015	16	Mild diarrhea	Ishikawa (36.595242,136.625671)	Pooled	2 (G3, G10)	Kobuvirus, Sapovirus
**Ishi-Ka4**	2015	16	Mild diarrhea	Ishikawa (36.595242,136.625671)	Pooled	1 (G3)	Kobuvirus, Picobirnavirus
**HgTa1**	2016	60	Without diarrhea	Tottori (35.503479,134.238266)	Single	1 (G2)	Astrovirus, Sapovirus, Picobirnavirus
**HgYa1-1**	2016	60	Without diarrhea	Tottori (35.503479,134.238266)	Single	1 (G4(	Sapelovirus, Astrovirus, Sapovirus, Rotavirus C
**Ishi-Ya2**	2016	23	Mild diarrhea	Ishikawa (36.595242,136.625671)	Single	1 (G1-PL-CP)	Kobuvirus
**Ishi-Ya3**	2016	24	Without diarrhea	Ishikawa (36.595242,136.625671)	Single	2 (G1-PL-CP, G9)	Kobuvirus
**Ishi-Ya4**	2016	24	Without diarrhea	Ishikawa (36.595242,136.625671)	Single	3 (G1-PL-CP, G3, G9)	-
**Ishi-Ka2**	2016	15	Without diarrhea	Ishikawa (36.595242,136.625671)	Single	1 (G?)	Kobuvirus
**Ishi-Ka3**	2016	20	Without diarrhea	Ishikawa (36.595242,136.625671)	Single	1 (G3)	-
**Ishi-Ka5**	2016	16	Without diarrhea	Ishikawa (36.595242,136.625671)	Single	1 (G3)	Kobuvirus, Rotavirus C
**Ishi-Ka6**	2016	16	Without diarrhea	Ishikawa (36.595242,136.625671)	Single	1 (G3)	Kobuvirus, Sapovirus, Rotavirus C
**Ishi-Ka7**	2016	16	Without diarrhea	Ishikawa (36.595242,136.625671)	Single	1 (G3)	-
**Ishi-Im8**	2016	11	Without diarrhea	Ishikawa (36.595242,136.625671)	Single	1 (G10)	Kobuvirus, Rotavirus A (GXP[23]), Posavirus
**Ishi-Im9**	2016	11	Without diarrhea	Ishikawa (36.595242,136.625671)	Single	1 (G10)	Rotavirus C, Rotavirus A (GXP[23]), Teschovirus, Kobuvirus, Picobirnavirus

**Table 2:** Summary of genomic information on EV-Gs isolated in the present study.

Strain name	Abbreviated name of strain	Genotype	Total reads	Enterovirus reads	Enterovirus reads (%)	Sequence length	DDBJ accession number
**EVG/Porcine/JPN/Iba464-3-1/2015**	Iba464-3-1	G1	1,819,610	8,572	0.5	7,372	LC316790
**EVG/Porcine/JPN/HgOg2-2/2015**	HgOg2-2	G1-PL-CP	369,006	1,329	0.4	7,995	LC316774
**EVG/Porcine/JPN/HgOg2-3/2015**	HgOg2-3	G1-PL-CP	1,067,294	1,304	0.1	7,995	LC316775
**EVG/Porcine/JPN/HgOg2-4-1/2015**	HgOg2-4-1	G1-PL-CP	399,976	2,610	0.7	8,004	LC316776
**EVG/Porcine/JPN/HgOg2-5/2015**	HgOg2-5	G1-PL-CP	267,732	2,001	0.7	7,982	LC316777
**EVG/Porcine/JPN/Iba464-4-1/2015**	Iba464-4-1	G1-PL-CP	2,016,670	6,134	0.3	8,003	LC316778
**EVG/Porcine/JPN/HgTa2-1-1/2015**	HgTa2-1-1	G1-PL-CP	685,940	5,841	0.9	8,007	LC316779
**EV/Porcine/JPN/HgTa2-2-1/2015**	HgTa2-2-1	G1-PL-CP	862,726	3,543	0.4	6,655	LC316780
**EVG/Porcine/JPN/HgTa2-5/2015**	HgTa2-5	G1-PL-CP	257,556	1,125	0.4	7,984	LC316781
**EVG/Porcine/JPN/MoI2-1-1/2015**	MoI2-1-1	G1-PL-CP	2,804,452	1,741	0.1	8,010	LC316782
**EVG/Porcine/JPN/MoI2-2-1/2015**	MoI2-2-1	G1-PL-CP	1,495,394	7,432	0.5	7,998	LC316783
**EVG/Porcine/JPN/MoI2-3-1/2015**	MoI2-3-1	G1-PL-CP	277,718	1,890	0.7	7,987	LC316784
**EVG/Porcine/JPN/IshiYa-5/2015**	Ishi-Ya5	G1-PL-CP	577,849	1,461	0.3	6,057	LC316785
**EVG/Porcine/JPN/Iba27-107/2015**	Iba27-107	G1-PL-CP	251,252	4,191	1.7	7,997	LC316786
**EVG/Porcine/JPN/Ishi-Ya2/2016**	Ishi-Ya2	G1-PL-CP	1,202,322	45,093	3.8	8,033	LC316787
**EVG/Porcine/JPN/Ishi-Ya3-1/2016**	Ishi-Ya3-1	G1-PL-CP	1,675,068	48,765	2.9	8,034	LC316788
**EVG/Porcine/JPN/Ishi-Ya4-1/2016**	Ishi-Ya4-1	G1-PL-CP	136,686	9,755	7.1	8,030	LC316789
**EVG/Porcine/JPN/Iba26-506/2014**	Iba26-506	G2	161,256	1,831	1.1	7,342	LC316792
**EVG/Porcine/JPN/HgOg2-4-2/2015**	HgOg2-4-2	G2	399,976	2,375	0.6	7,365	LC316793
**EVG/Porcine/JPN/HgTa1/2016**	HgTa1	G2	1,405,590	1,627	0.1	7,347	LC316794
**EVG/Porcine/JPN/HgYa2-1/2015**	HgYa2-1	G2-PL-CP	1,373,440	11,521	0.8	8,016	LC316791
**EVG/Porcine/JPN/Bu3-4/2014**	Bu3-4	G3	582,500	437,580	75.1	7,397	LC316795
**EVG/Porcine/JPN/Bu3-5/2014**	Bu3-5	G3	639,686	3,698	0.6	7,360	LC316796
**EVG/Porcine/JPN/Bu3-6/2014**	Bu3-6	G3	585,342	5,032	0.9	7,374	LC316797
**EVG/Porcine/JPN/Bu3-7/2014**	Bu3-7	G3	797,002	13,498	1.7	7,378	LC316798
**EVG/Porcine/JPN/Bu4-1/2014**	Bu4-1	G3	467,920	6,547	1.4	7,381	LC316799
**EVG/Porcine/JPN/Bu4-2/2014**	Bu4-2	G3	629,719	5,821	0.9	7,355	LC316800
**EVG/Porcine/JPN/Bu4-4/2014**	Bu4-4	G3	957,684	4,258	0.4	7,355	LC316801
**EVG/Porcine/JPN/Bu4-6/2014**	Bu4-6	G3	446,798	68,970	15.4	7,381	LC316802
**EVG/Porcine/JPN/Bu5-1/2014**	Bu5-1	G3	609,055	36,635	6.0	7,390	LC316803
**EVG/Porcine/JPN/Bu5-6/2014**	Bu5-6	G3	666,801	33,387	5.0	7,385	LC316804
**EVG/Porcine/JPN/Bu6-5/2014**	Bu6-5	G3	432,420	37,095	8.6	7,384	LC316805
**EVG/Porcine/JPN/Bu8-2/2014**	Bu8-2	G3	622,936	2,334	0.4	7,377	LC316806
**EVG/Porcine/JPN/Bu8-4/2014**	Bu8-4	G3	569,460	1,929	0.3	6,427	LC316807
**EVG/Porcine/JPN/Ishi-Sa5/2015**	Ishi-Sa5	G3	2,389,638	2,845	0.1	7,350	LC316808
**EVG/Porcine/JPN/Ishi-Ka3-1/2015**	Ishi-Ka3-1	G3	2,648,440	1,868,538	70.6	7,396	LC316809
**EVG/Porcine/JPN/Ishi-Ka4/2015**	Ishi-Ka4	G3	597,344	4,130	0.7	7,366	LC316810
**EVG/Porcine/JPN/Ishi-Sa4/2015**	Ishi-Sa4	G3	1,130,432	1,345	0.1	7,363	LC316811
**EVG/Porcine/JPN/Ishi-Ka3/2016**	Ishi-Ka3	G3	326,014	3,682	1.1	7,367	LC316812
**EVG/Porcine/JPN/Ishi-Ka5-1/2016**	Ishi-Ka5-1	G3	442,360	516	0.1	7,303	LC316813
**EVG/Porcine/JPN/Ishi-Ka6/2016**	Ishi-Ka6	G3	536,200	27,564	5.1	7,383	LC316814
**EVG/Porcine/JPN/Ishi-Ka7/2016**	Ishi-Ka7	G3	903,384	1,524	0.2	7,296	LC316815
**EVG/Porcine/JPN/Ishi-Ya4-2/2016**	Ishi-Ya4-2	G3	136,686	11,960	8.7	7,357	LC316816
**EVG/Porcine/JPN/Iba464-3-2/2015**	Iba464-3-2	G4	1,819,610	9,165	0.5	7,356	LC316817
**EVG/Porcine/JPN/HgYa1-1/2016**	HgYa1-1	G4	1,083,038	319	0.03	5,057	LC316818
**EVG/Porcine/JPN/HgTa2-1-2/2015**	HgTa2-1-2	G6	685,940	2,108	0.3	7,344	LC316819
**EVG/Porcine/JPN/Iba464-4-2/2015**	Iba464-4-2	G6	2,016,670	1,596	0.1	7,341	LC316820
**EVG/Porcine/JPN/HgTa2-2-2/2015**	HgTa2-2-2	G9	862,726	2,830	0.3	7,354	LC316821
**EVG/Porcine/JPN/Iba27-21/2015**	Iba27-21	G9	298,500	823	0.3	7,266	LC316822
**EVG/Porcine/JPN/Iba26-489/2014**	Iba26-489	G9	130,796	13,406	10.2	7,373	LC316823
**EVG/Porcine/JPN/Iba27-20/2015**	Iba27-20	G9	103,974	4,380	4.2	7,365	LC316824
**EVG/Porcine/JPN/Ishi-Ya3-2/2016**	Ishi-Ya3-2	G9	1,675,068	46,365	2.8	7,355	LC316825
**EVG/Porcine/JPN/Ishi-Ya4-3/2016**	Ishi-Ya4-3	G9	136,686	12,043	8.8	7,300	LC316826
**EVG/Porcine/JPN/HgYa2-3-1/2015**	HgYa2-3-1	G10	722,010	3,127	0.4	7,330	LC316827
**EVG/Porcine/JPN/HgYa2-4-1/2015**	HgYa2-4-1	G10	1,344,284	1,881	0.1	7,332	LC316828
**EVG/Porcine/JPN/Ishi-Ka3-2/2015**	Ishi-Ka3-2	G10	2,648,440	1,868,538	70.6	7,345	LC316829
**EVG/Porcine/JPN/Ishi-Im8/2016**	Ishi-Im8	G10	1,414,912	102,556	7.2	7,382	LC316830
**EVG/Porcine/JPN/Ishi-Im9-1/2016**	Ishi-Im9-1	G10	1,759,264	1,997	0.1	7,040	LC316831
**EVG/Porcine/JPN/Ishi-Ka2/2016**	Ishi-Ka2	G?	960,736	1,546	0.2	7,360	LC316832

### Phylogenetic analysis and pairwise identity evaluation of the *VP1* gene

**Fig 1:**
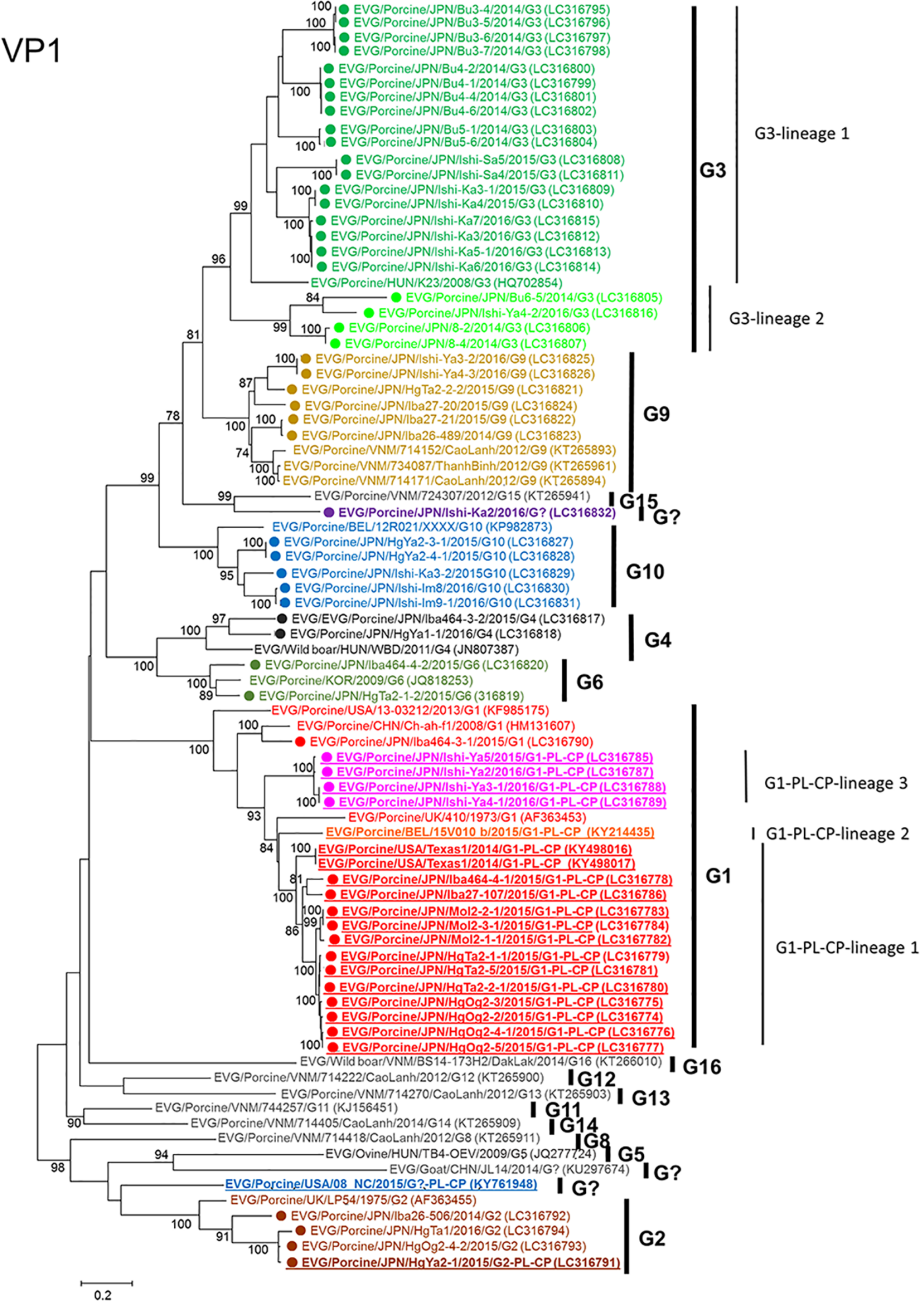
A phylogenetic tree of complete *VP1* coding-region sequences. Phylogenetic analyses based on nt sequences of the full-length *VP1* coding region of 59 EV-Gs detected in this study was performed using reference strains from the DDBJ/EMBL/GenBank databases. The host, country of origin, strain name, and year of detection are shown for each strain. DDBJ/EMBL/GenBank accession numbers are indicated in parentheses. Phylogenetic trees that were constructed by the maximum likelihood method in MEGA 5.22 with bootstrap values (1000 replicates) above 70 are presented. The bar represents a corrected genetic distance. The genotypes are indicated on the right-hand side. ● denotes EV-G strains detected in this study. EV-G PL-CP strains are indicated with underlined bold text.

Because EV-G discrimination is based on sequence identities of the complete *VP1* gene (http://www.picornaviridae.com/), a phylogenetic tree using the complete *VP1* nt sequence was constructed. Japanese EV-G strains clustered with the reference strains of G1 (17 strains), G2 (four strains), G3 (22 strains), G4 (two strains), G6 (two strains), G9 (six strains), and G10 (five strains). One strain named Ishi-Ka2 branched independently and did not cluster with any reference strains (Fig 1). Because >25% difference in complete *VP1* nt sequences between isolates is a criterion for genotype classification [13–14], a pairwise comparison of complete *VP1* nt sequences was conducted (Table 3 and S1 Table). Bu6-5, Bu8-2, Bu8-4, and Ishi-Ya4-2 formed one cluster but were found to be slightly related to the G3 group. Although complete *VP1* nt sequence identities of these strains to those of other G3 strains (except for Ishi-Ka3, Ishi-Ka3-1, Ishi-Ka4, Ishi-Ka5-1, Ishi-Ka6, and Ishi-Ka7) were <75.0% (69.8%-74.9%), these four strains shared ≥75.0% nt sequence identities with Ishi-Ka3, Ishi-Ka3-1, Ishi-Ka4, Ishi-Ka5-1, and Ishi-Ka6 (S1 Table). Therefore, we tentatively classified these strains as G3-lineage 2 (Fig 1). Ishi-Ka2 revealed low nt sequence identities (57.5% to 73.1%) with other genotypes and thus Ishi-Ka2 may represent a new serotype of EV-G (Table 3).

**Table 3:** Pairwise nt (lower left) and aa (upper right) sequence identity levels for VP1 between genotypes of EV-Gs.

	G1	G2	**G3**	**G4**	**G5**	**G6**	**G8**	**G9**	**G10**	**G11**	**G12**	**G13**	**G14**	**G15**	**G16**	**G? (08NC)**	**G? (JL14)**	**G? (Ka2)**
**G1**	79.5–100 71.0–100	59.2–64.8	59.1–65.7	64.0–70.0	56.1–61.1	63.4–69.4	58.8–61.6	60.4–66.7	62.8–68.8	66.8–71.7	63.4–66.6	59.2–63.4	68.0–72.9	60.7–63.9	62.2–65.0	58.1–63.0	54.4–58.3	60.8–64.7
**G2**	58.6–64.0	93.3–99.7 77.5–98.6	56.6–61.2	62.7–65.5	70.9–71.9	64.2–65.3	68.3–70.8	58.6–61.5	61.4–63.5	68.3–69.4	65.1–65.5	60.9–62.0	66.9–68.0	62.1–64.2	60.2–61.3	76.1–76.4	66.3–67.0	60.8–62.2
**G3**	58.2–64.7	57.5–61.9	81.4–100 69.8–100	63.2–67.4	53.3–57.3	65.7–69.6	55.9–60.7	78.5–83.2	71.9–77.5	67.0–69.0	66.2–72.9	65.3–68.4	64.9–69.0	75.4–80.0	60.4–64.9	57.0–61.2	54.4–57.3	75.1–79.3
**G4**	62.9–67.4	61.3–65.0	60.4–66.1	91.2–94.7 77.3–80.6	64.6–64.9	79.6–81.0	61.3–62.7	65.6–68.1	67.7–69.5	70.3–71.0	66.2–66.9	65.9–66.9	68.0–69.4	68.1–70.9	66.4–67.5	63.7–64.8	60.0–61.1	68.5–68.9
**G5**	58.3–60.2	64.0–65.4	55.5–58.7	61.5–62.3	-	62.6–64.3	66.0	59.1–60.1	59.8–60.8	65.3	64.9	61.1	63.5	58.7	60.7	71.6	68.8	57.1
**G6**	60.7–67.6	61.5–64.1	62.5–66.6	70.5–73.0	60.8–61.9	96.8–98.6 83.3–85.2	62.1–62.3	68.9–71.3	68.5–70.3	72.2–72.9	69.8–71.2	67.0–68.1	68.8	70.2	66.2–66.9	63.9–64.2	58.0–59.4	67.5–68.9
**G8**	57.5–62.3	64.7–66.7	58.5–61.7	61.9–64.2	61.6	62.0–62.5	-	57.9–59.7	60.4–62.5	69.0	66.6	65.1	66.6	60.0	60.1	71.1	63.2	61.2
**G9**	60.0–64.4	57.7–61.2	68.8–75.5	62.2–66.0	57.8–59.8	63.4–67.3	58.1–60.1	93.7–100 81.5–99.9	77.7–81.3	70.1–71.5	70.4–72.3	66.9–69.4	68.0–69.4	81.3–82.4	64.1–65.3	59.7–62.2	60.8–62.0	78.6–81.1
**G10**	60.8–65.4	60.9–63.0	66.1–70.5	63.7–66.8	57.7–59.4	62.7–65.5	59.0–62.0	69.4–74.2	92.3–100 79.0–100	69.4–70.4	69.7–71.5	67.6–69.0	69.4–70.4	75.4–76.1	66.2–66.6	61.8–64.9	58.7–59.4	73.3–76.1
**G11**	63.1–67.3	65.3–66.2	64.2–66.6	64.9–69.9	62.7	67.6–68.2	66.1	65.7–67.8	66.1–68.0	-	75.3	69.6	76.7	66.9	68.1	69.0	62.5	67.0
**G12**	62.3–66.0	61.4–63.7	63.3–66.7	64.4–65.1	62.0	65.3–65.5	64.2	65.4–66.9	65.3–66.7	68.3	-	74.8	73.1	68.3	66.0	68.7	63.5	67.4
**G13**	61.4–64.4	60.3–61.6	61.3–65.4	63.4–64.3	60.8	63.4–63.6	63.6	63.2–65.4	62.9–65.0	64.9	68.8	-	67.8	66.9	66.6	63.4	59.3	65.3
**G14**	63.3–67.6	63.6–65.0	61.5–65.5	66.4–67.5	60.5	65.2–66.7	66.1	64.3–66.8	64.3–65.3	72.2	68.2	63.5	-	67.6	68.9	65.9	61.8	70.2
**G15**	58.8–62.1	61.2–62.2	68.0–69.7	65.2–66.7	59.1	63.3–64.8	61.9	68.5–70.2	67.8–69.3	64.8	64.3	62.3	62.3	-	64.7	61.1	58.4	85.3
**G16**	58.8–62.2	56.1–58.9	56.5–60.8	60.0–62.0	55.7	61.3–62.0	57.1	58.5–61.6	60.3–61.6	63.2	62.2	60.9	62.5	59.3	-	63.0	58.6	64.9
**G? (08NC)**	58.3–61.4	68.1–69.1	56.8–61.3	60.7–64.1	64.2	60.6–61.9	68.1	60.1–61.3	60.1–61.8	67.7	64.0	62.7	66.6	60.1	56.6	-	67.7	62.6
**G? (JL14)**	53.9–57.9	61.4–64.0	54.9–58.5	58.3–59.8	64.3	56.6–57.6	59.9	58.9–60.3	57.3–58.2	60.1	60.0	56.7	60.0	58.7	56.3	64.1	-	57.8
**G? (Ka2)**	59.6–63.4	60.6–62.4	66.0–71.8	63.4–64.9	58.0	62.7–63.8	61.7	68.9–72.5	68.1–70.4	64.9	63.6	63.3	64.9	73.3	58.3	60.7	57.3	-

### Identification and genomic characterization of EV-Gs with a PL-CP sequence

**Fig 2:**
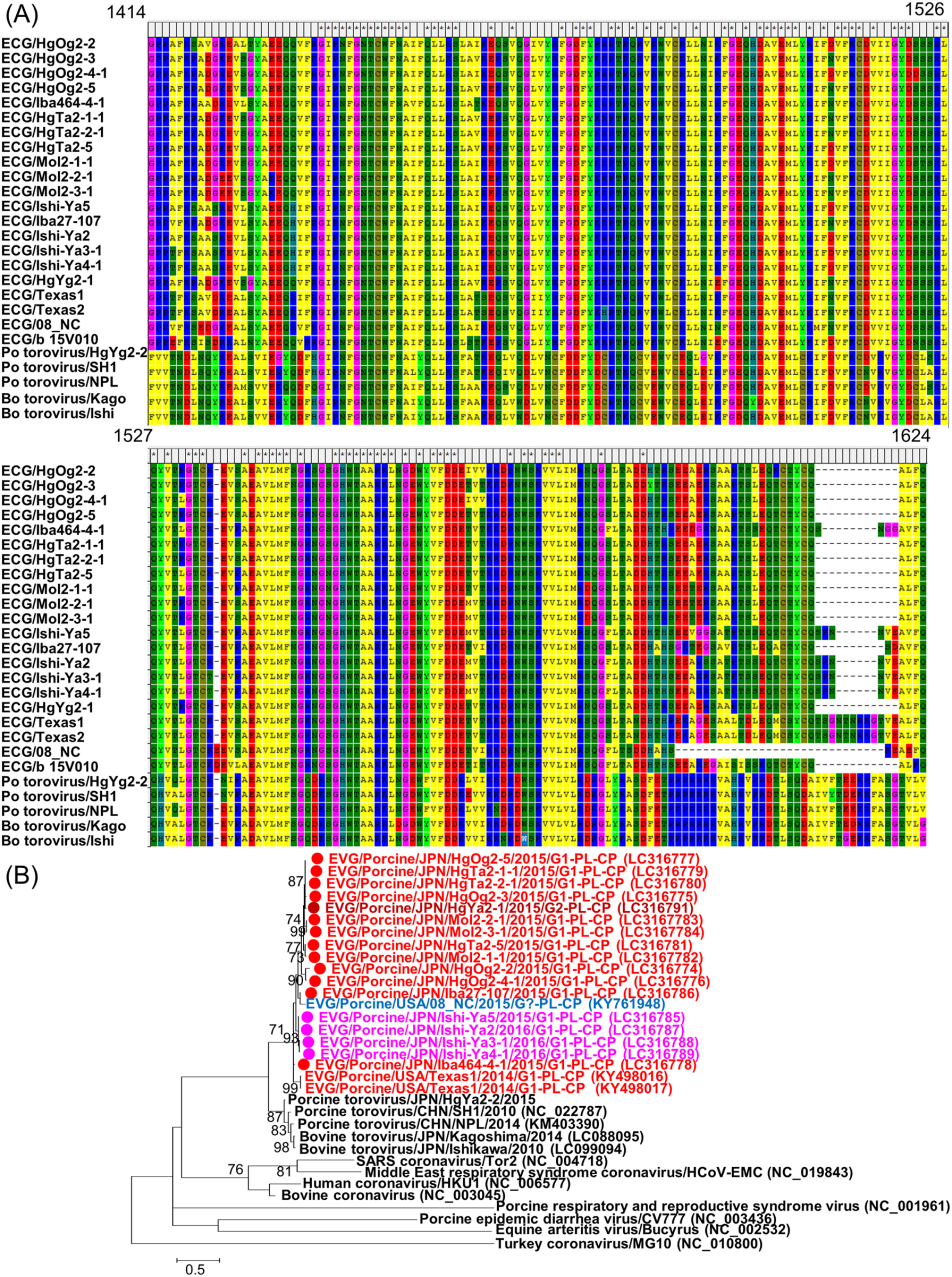
Amino acid (aa) sequence comparison of EV-G PL-CP with that of torovirus PL-CP. (A) Alignment of aa sequences of EV-G PL-CP inserted between regions 2C and 3A with PL-CPs of porcine torovirus and bovine torovirus. (B) Phylogenetic analyses based on aa sequences of EV-G PL-CP and PL-CP of nidoviruses including toroviruses. Phylogenetic trees that were constructed by the maximum likelihood method in MEGA 5.22 with bootstrap values (1000 replicates) above 70 are shown. The scale bar indicates nucleotide substitutions per site. ● denotes EV-G strains detected in this study.

Complete or nearly complete aa sequences of the coding-sequence region (CDS) of 59 Japanese EV-G strains were aligned and compared. We found that 17 of the 59 strains contain extra 633 to 651 nt (211 to 217 aa) within the 2C-3A coding region. According to BLAST analysis, these sequences have sequence homology to the PL-CP sequence variants that were recently identified in EV-G strains from the USA and Belgium [24–26]. Each inserted sequence is located between the coding regions 2C and 3A as in the USA and Belgium strains. The insertion sequences were aligned and compared with those of the PL-CP of EV-G strains from the USA and Belgium and with the PL-VP sequences in the genome of nidoviruses including porcine and bovine toroviruses by phylogenetic analysis and pairwise sequence comparison (Fig 2 and S2 Table). The PL-CP sequence of Japanese EV-G1 and that of Japanese EV-G2 revealed ≥74.0% nt and ≥74.6% aa sequence identities to each other and to the USA and Belgium EV-Gs and clustered in one group but are distantly related to those of porcine and bovine toroviruses, showing lower sequence identities (57.0% to 64.6% in the nt sequence and 49.6% to 58.7% in the aa sequence). EV-G HgYa2-1 and porcine torovirus HgYa2-2 were identified on the same farm at the same time; however, the nt and aa sequence identity between the PL-CP sequences of those strains was 62.3% and 54.3%, respectively (S2 Table). Japanese EV-G strains carrying PL-CP were subdivided into G1-PL-CP lineage 1, G1-PL-CP lineage 2, and G2 in the VP1 phylogenetic tree (Fig 1); however, these groups were not clearly detectable in the PL-CP tree (Fig 2)

### Phylogenetic analysis and similarity plot evaluation for the nearly full genome of EV-Gs

**Fig 3:**
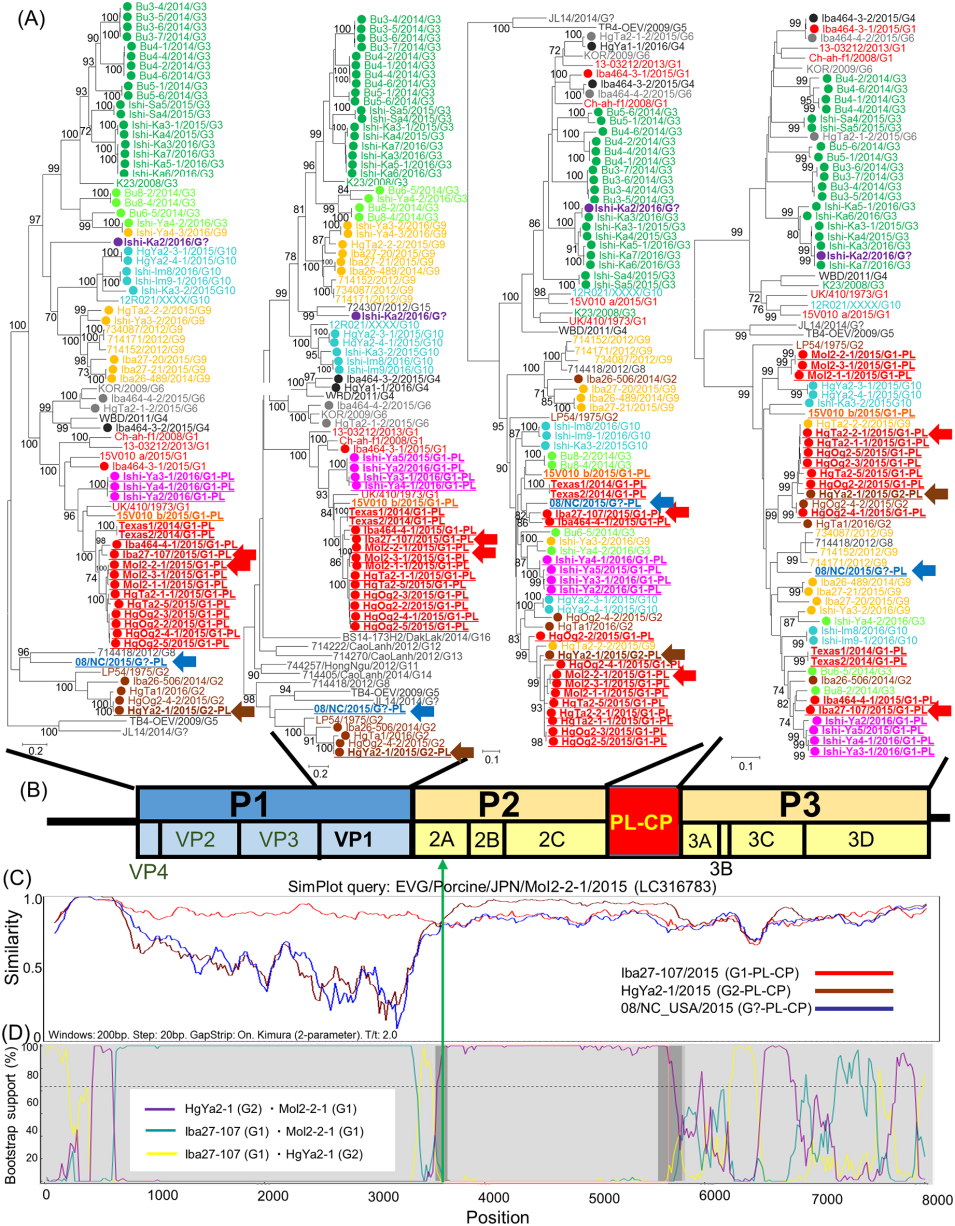
Whole-genome analysis of EV-Gs by means of phylogenetic trees, SimPlot, and RDP. (A) Phylogenetic analyses based on nt sequences of VP4-VP3, VP1, P2, and P3 of 59 EV-Gs detected in this study, using reference strains from DDBJ/EMBL/GenBank databases. An abbreviated strain name, year of detection, and genotype are presented for each strain. Phylogenetic trees that were constructed by the maximum likelihood method in MEGA 5.22 with bootstrap values (1000 replicates) above 70 are shown. The bar represents a corrected genetic distance. The genotypes are indicated on the right-hand side. ● denotes EV-G strains detected in this study. EV-G PL-CP strains are indicated with underlined bold text. (B) Genome structure of EV-G. (C) Similarity plots of the entire genomes of EV-G1-PL-CP Iba27-107 (red curve), EV-G2-PL-CP HgYa2-1 (brown curve), EV-G?-PL-CP 08/NC (blue curve), and EV-G1-PL-CP MoI2-2-1 as query sequences, with a sliding window of 200 nt and a moving step size of 20 nt. (D) Recombination breakpoint analysis of EV-G2-PL-CP HgYa2-1 vs. EV-G1-PL-CP MoI2-2-1 (purple curve), EV-G1-PL-CP Iba27-107 vs. EV-G1-PL-CP MoI2-2-1 (blue curve), and EV-G1-PL-CP Iba27-107 vs. EV-G2 HgYa2-1 (yellow curve).

To further investigate the genomic relations among EV-G strains, phylogenetic trees based on nt sequences of three regions (VP4-VP3, VP1, P2, and P3) were constructed. The tree for VP4-VP3 was similar to that of VP1, but the P2 and P3 trees showed topologies different from each other and no clear-cut EV-G types could be defined (Fig 3A). EV-G1-PL-CP lineage 3 was found to be related to G1-PL-CP lineage 2 and 3 in the trees for VP4-VP3 and VP1, whereas G1-PL-CP lineage 3 was closely related to G3 and G9 strains in the P2 tree and to G3 and G1-PL-CP strains in the P3 tree. The G2-PL-CP strain HgYa2-1 showed high homology to G2 strains in the tree for VP4-VP3 and VP1, whereas HgYa2-1 showed high similarity with G1-PL-CP lineage 1 strains in regions P2 and P3 (Fig 3A). By SimPlot analysis, the crossover site was mapped to the 2A region. The G2-PL-CP strain HgYa2-1 revealed that the downstream region of the crossover site has high similarity to the G1-PL-CP strain MoI2-2-1 (Fig 3C). To find a possible recombination breakpoint, a bootstrap scanning analysis was conducted. A possible recombination breakpoint was identified in the middle of the 2A region (Fig 3D).

Ishi-Ka2 branched independently in the trees for VP4-VP3 and VP1, whereas Ishi-Ka2 clustered with G3-lineage 1 and formed a cluster with G3 strains identified on the same farm (S1A Fig). SimPlot analysis suggested that Ishi-Ka2 has extremely high similarity to G3-lineage 1 strain Ishi-Ka7 in regions 2C and P3 (S1C Fig).

G3-lineage 2 strains showed a topology similar to that of the VP1 tree in VP4-VP3; however, the G3-lineage 2 strains branched separately from the G3-lineage-1 strains in the P2 and P3 trees (S1A Fig) and were found to be closely related to each other throughout the genome (S1D Fig).

## Discussion

Although we did not initially aim to determine EV-G prevalence among pigs in Japan in this study, contigs that were longer than 5,000 nt were found in 22.5% (50/222) of pigs and on 23.4% (18/77) of farms, suggesting that EV-Gs are widespread among Japanese pigs. Forty-four strains out of 59 (74.6%) were detected in healthy pigs, indicating that EV-Gs seem not to be associated with diarrhea in pigs, in accordance with other reports [13–14, 38]. Because the detection limit of the method was not tested, a true prevalence study is needed in the future.

EV-G genotyping is based on >25% divergence between VP1 nucleotide sequences [14, 39]. In the present study, according to the criteria, seven genotypes (G1, G2, G4, G6, G9, G10, and G?) were found in the feces samples from Japanese pigs, and the predominant genotypes were G3 (37.3%; 22/59) and G1 (28.8%; 17/59; Table 1, Fig 1). There are few studies on the genotyping of EV-Gs in pigs, and limited information is available from DDBJ/EMBL/GenBank databases. G1 and G6 types are predominant genotypes in Vietnam, whereas G3, G2, and G4 types appear to be common genotypes in Spain (however, that study did not analyze complete *VP1* sequence) [40]. To date, G1–G16 genotypes and at least three EV-Gs with an unassigned genotype, including Ishi-Ka2 in this study, have been reported [13–14, 25, 41]. Owing to the limited number of reports on a specific geographic area, probably not all genotypes of EV-Gs are known at present. Further studies are needed for a comprehensive understanding of the genetic diversity of EV-Gs in other geographic areas.

Picornaviruses show significant genetic variability driven by both mutations and recombination events [42–43]. Ishi-Ka2 manifested >25% VP1 nucleotide sequence divergence from other strains; therefore, Ishi-Ka2 can be considered a new serotype of EV-Gs. Nonetheless, Ishi-Ka2 shares high sequence homology with the G3-lineage 1 strain Ishi-Ka7, which was identified in a pig kept on the same farm, except for the P1 region. It is likely that Ishi-Ka2 emerged by possible recombination events; however, the putative recombination points could not be identified, and the origins of these recombination events are unclear because the recombination counterparts of these strains could not be found in the DDBJ/EMBL/GenBank databases or our dataset. G3-lineage 2 strains have sequence homology to G3-lineage 1 in the P1 region, but they are distantly related to G3-lineage 1 on the basis of regions P2 and P3. Because VP1 induces a host immune response, serological properties can be hypothesized based on sequence homology of the *VP1* gene. On the other hand, these results suggest that full genome analysis may be needed in addition to the genotyping approaches based solely on the *VP1* gene for precise EV-G classification.

RNA recombination events contribute to genetic diversity and may lead to changes in virulence, escape from host immunity, and adaptation to a new host [42–50]. EV-G strains carrying PL-CP in pigs with diarrhea have been reported in the USA and Belgium [24–26]. In these cases, EV-G-PL-CPs were detected solely or with low abundance of PEDV. Shang et al. constructed an infectious clone of the EV-G-PL-CP strain, 08/NC_USA/2015, and compared it with a PL-CP knockout recombinant virus. They found that the PL-CP knockout virus showed impaired growth and induced higher expression levels of innate-immunity genes, suggesting that EV-G-PL-CP strains acquire pathogenesis via a recombination event [25]. Four out of 17 Japanese EV-G-PL-CP strains were detected in diarrheic cases of pigs; however, 13 EV-G-PL-CP strains were isolated from healthy pigs. In all cases of detection of EV-G-PL-CP in Japan, EV-G-PL-CP strains were identified together with other enteric viruses, such as astrovirus, sapelovirus, posavirus, rotavirus, picobirnavirus, sapovirus, teschovirus, torovirus, PEDV, St-Valerien virus, or kobuvirus (Table 1). Mixed infection with EV-G-PL-CP and other enteric viruses may influence the pathogenicity of EV-G-PL-CP strains.

The sequences of PL-CP of Japanese EV-G PL-CP strains are distantly related to the sequences derived from ORF1 of toroviruses, even though they were simultaneously identified on the same farm, and they have homology to those of USA and Belgium strains (Fig 2), suggesting that a recombination event between an EV-G and torovirus occurred in the past. By recombination analyses, possible recombination events between EV-G-PL-CP strains were uncovered and a recombination breakpoint was identified in the middle of region 2A (Fig 3), in agreement with another report that describes a recombinant event between EV-G8 and EV-G9 [14], suggesting that this point may be a hotspot of recombination events of EV-G. Furthermore, VP1-2A junction is a known recombination hot-spot in human enteroviruses and this was discussed in many papers [51–55]. The present recombination profile in EV-G described here apparently mirrors that in human enteroviruses. EV-Gs that received PL-CP have been evolving independently and gaining genetic diversity via recombination events.

## Conclusions

By a metagenomics approach, high genetic diversity of EV-Gs, including new genotypes and high prevalence of EV-Gs carrying PL-CP, was observed among EV-G isolates from the feces of Japanese pigs. EV-Gs comingle and pose a risk of coinfection in the current growing and high-density pig husbandry system of Japan. Coinfection of a single animal with multiple EV-Gs, including EV-G-PL-CP strains, may lead to recombination events, which may in turn promote genetic diversity of EV-Gs and EV-G-PL-CPs. These findings may improve our understanding of the molecular epidemiology and evolution of EV-Gs.

## Supporting information

S1 Fig Whole-genome analysis of EV-G isolates using a phylogenetic tree and SimPlot. (A) Phylogenetic analyses based on nt sequences of VP4-VP3, VP1, P2, and P3 of 59 EV-Gs detected in this study with reference strains from DDBJ/EMBL/GenBank databases. An abbreviated strain name, year of detection, and genotype are shown for each strain. Phylogenetic trees that were constructed by the maximum likelihood method in MEGA 5.22 with bootstrap values (1000 replicates) above 70 are presented. The bar represents a corrected genetic distance. The genotypes are shown on the right-hand side. ● denotes EV-G strains detected in this study. EV-G PL-CP strains are indicated with underlined bold text. (B) Genome structure of EV-G. (C, D) Similarity plots of the entire genomes of EV-G3-lineage 1 strains (green curve), EV-G3-lineage 2 strains (light green curve), EV-G? Ishi-Ka2 (purple curve), and EV-G3-lineage 1 Ishi-Ka7 (C) and EV-G3-lineage 2 Ishi-Ya4-2 (D) as query sequences, with a sliding window of 200 nt and a moving step size of 20 nt.
(TIF)

S1 Table Pairwise nucleotide (lower left) and amino acid (upper right) sequence identities (%) of completeVP1 gene between Japanese EV-Gs and other EV-G strains. (XLSX)

S2 Table Pairwise nucleotide (lower left) and amino acid (upper right) sequence identities (%) of the PL-CP between EV-G strains and porcine and bovine toroviruses. (XLSX)

S3 Table Information of co-infection with other viruses. (XLSX)
