# Supplementary figures and images for: Genetic diversity and recombination of enterovirus G strains in Japanese pigs: High prevalence of strains carrying a papain-like cysteine protease sequence in the enterovirus G population
Source: PLoS One. 2018 Jan 11;13(1):e0190819. doi: 10.1371/journal.pone.0190819 (PMC5764308; doi:10.1371/journal.pone.0190819)

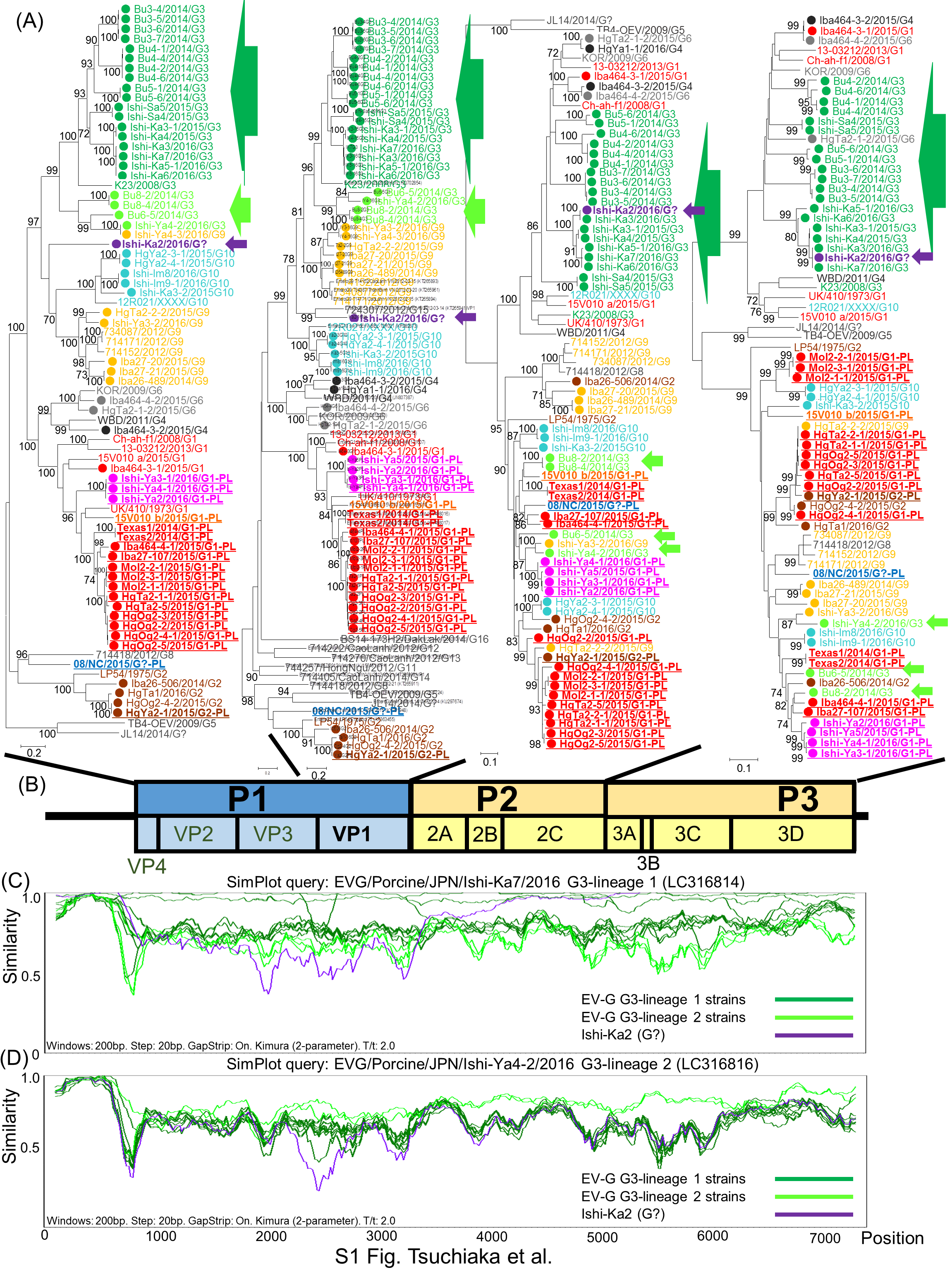

Supplement: S1 Fig — (A) Phylogenetic analyses based on nt sequences of VP4-VP3, VP1, P2, and P3 of 59 EV-Gs detected in this study with reference strains from DDBJ/EMBL/GenBank databases. An abbreviated strain name, year of detection, and genotype are shown for each strain. Phylogenetic trees that were constructed by the maximum likelihood method in MEGA 5.22 with bootstrap values (1000 replicates) above 70 are presented. The bar represents a corrected genetic distance. The genotypes are shown on the right-hand side. ● denotes EV-G strains detected in this study. EV-G PL-CP strains are indicated with underlined bold text. (B) Genome structure of EV-G. (C, D) Similarity plots of the entire genomes of EV-G3-lineage 1 strains (green curve), EV-G3-lineage 2 strains (light green curve), EV-G? Ishi-Ka2 (purple curve), and EV-G3-lineage 1 Ishi-Ka7 (C) and EV-G3-lineage 2 Ishi-Ya4-2 (D) as query sequences, with a sliding window of 200 nt and a moving step size of 20 nt. (TIF) [file pone.0190819.s001.tif]
